# Participation of WNT and *β*-Catenin in Physiological and Pathological Endometrial Changes: Association with Angiogenesis

**DOI:** 10.1155/2015/854056

**Published:** 2015-08-20

**Authors:** Jolanta Kiewisz, Tomasz Wasniewski, Zbigniew Kmiec

**Affiliations:** ^1^Department of Histology and Embryology, Faculty of Medical Sciences, University of Warmia and Mazury, Warszawska 30, 10-082 Olsztyn, Poland; ^2^Department of Obstetrics and Gynecology, Faculty of Medical Sciences, University of Warmia and Mazury, Zołnierska 18, 10-561 Olsztyn, Poland

## Abstract

WNT proteins are involved in embryonic development, sex determination, stem cell recruitment, angiogenesis, and cancer. They take part in morphological changes in the endometrium during development, regulate processes of endometrial proliferation and differentiation. This review presents current knowledge about implication of WNT proteins and *β*-catenin in physiological endometrial functions as well as their involvement in uterine carcinogenesis. Influence of WNT proteins on the formation of blood vessel, taking place both under healthy and pathological conditions, is also considered. Participation of WNT proteins, *β*-catenin, and inhibitors and inducers of WNT signaling in the process of endometrial angiogenesis is largely unknown. Thus, confirmation of their local and systemic participation in the process of endometrial angiogenesis may in the long term help to establish new diagnostic and therapeutic approaches in conditions associated with the pathology of the female reproductive system.

## 1. Introduction

WNT are proteins involved in physiological and pathological processes such as embryonic development, sex determination, malignant transformation, endothelial cell differentiation, and angiogenesis [1, 2]. Angiogenesis is required for formation and remodeling of the endometrial vascular system in the course of menstrual cycle. Impaired process of differentiation of endometrial epithelium may lead to cancer, usually accompanied by pathological angiogenesis.

In this review, we present data about implication of WNT proteins and *β*-catenin in physiological endometrial functions as well as their involvement in uterine carcinogenesis. We also present current knowledge about influence of WNT proteins on the formation of blood vessel, both under healthy and pathological conditions. The proposed hypothesis about participation of WNT proteins in the regulation of the formation of microvessels might provide a conceptual framework for the design of future experiments.

## 2. General Characteristics of* WNT* Genes and Proteins

*Wingless* gene is responsible for segmentation during embryogenesis and legs formation during transformation of* Drosophila melanogaster*. Homologous gene* int-1*, detected in mammals, becomes activated upon cell integration of the MMTV virus (*mouse mammary tumor virus*), which causes mammary tumors in mice. Comparison of the amino acid sequences of the proteins encoded by these two genes showed their high homology, while combination of the abbreviations of gene names gives the name for the whole gene family,* WNT *(*WNT* =* W*g + I*NT*). To date, the largest number (19) of* WNT* genes was found in mice and humans. Their existence was also confirmed in the nematode* Caenorhabditis elegans*, zebrafish (*Danio rerio*), amphibians of the* Xenopus* genus, and chicken (*Gallus gallus domesticus*). Based on their capability to induce the malignant transformation in cell lines derived from murine mammary gland epithelial cells (C57MG),* WNT* genes were divided into two groups. The first group includes genes encoding cysteine-rich, secretory glycoproteins with oncogenic characteristics: WNT1, WNT3, WNT3A, WNT7, WNT8, and WNT8B, while the other encodes proteins lacking the properties to induce malignant transformation: WNT2, WNT4, WNT5A, WNT5B, WNT6, WNT7B, and WNT11 [2]. Hydrophobic nature and activity of these proteins were associated with binding of cysteine residues with palmitic acid.

WNT proteins activate the canonical (genes involved in the malignant transformation) and noncanonical (genes not involved in the malignant transformation) cell signaling pathways. Canonical signaling pathway known also as WNT/*β*-catenin signaling pathway is activated by WNT proteins joined with the complex of Frizzled (FZD)/low-density-lipoprotein receptor-related protein (LRP) on the cell surface. Activated cytoplasmic protein called Dishevelled (DSH) inhibits the activity of a protein APC/GSK3*β* complex (Axin/Adenomatous Polyposis Coli/Glycogen Synthase Kinase 3*β*) responsible for the degradation of *β*-catenin. After stimulation with WNT, cytoplasmic *β*-catenin is translocated to the nucleus, where it activates transcription factors: T cell factor (TCF) and lymphoid enhancer factor (LEF). These transcription factors change the expression level of target genes encoding proteins implicated in cell proliferation and survival (cyclin D1, c-Myc), cellular migration (CD44), cell adhesion (CDH1), digestion of extracellular matrix (MMP7), and many others [3]. Because *β*-catenin binds to *α*-catenin and cytoplasmic domain of E-cadherin, one of the methods of inhibiting cell signal induced by WNT is to increase the level of E-cadherin. In mammals, the activation of the WNT/*β*-catenin pathway leads to the enhanced recruitment of stem cells and amplification of their pluripotency.

The other group of WNT which act through *β*-catenin-independent pathway can activate FZD receptor family. The initiation of signal cascades results in the release of calcium ions, activation of protein kinase C (PKC) and calcium-calmodulin dependent protein kinase II (CAMKII). It has been established that the WNT/Ca^2+^ signal transduction pathway antagonizes the action of WNT/*β*-catenin signaling [4] and may be associated with cell proliferation and migration [5]. In the second type of signaling pathway, the planar cell polarity pathway and FZD receptors through DSH protein activate small G proteins, Rac and Rho kinases, and c-Jun N-terminal kinase (JNK). This leads to the restructuring of the cytoskeleton proteins, migration of the cell, and the acquisition of cell polarity [2].

WNT-mediated signaling pathways can be modulated through secreted Frizzled-related proteins (sFRP) and Dickkopf (DKK) proteins. sFRP proteins bind directly to WNT proteins, while DKK proteins block LRP5/6 coreceptors. In both cases, WNT ability for signal transduction is blocked [2].

## 3. *WNT* Genes and Proteins in the Endometrial Physiology 

### 3.1. WNT Proteins in the Female Reproductive Tract Development

Developmental changes of the endometrium are mainly associated with the expression of* WNT4*,* WNT5A*, and* WNT7A* genes as demonstrated in mouse [6–8] and pig [9, 10]. However,* WNT4*,* WNT5A*, and* WNT7A* genes expression was presented also in developed uterus in humans [11, 12], sheep [13], horse [14], and pig [9, 10, 15, 16].

*Wnt4* gene is expressed in the primordial gonads of mouse embryos [17] and Wnt4 protein influences the process of gametogenesis [18]. During mouse embryonic development,* Wnt4* gene is expressed in stromal cells of the forming endometrium [19]. In mice lacking* Wnt4* gene, sex reversion, partial atrophy of the Müllerian ducts, masculinization, and morphological and functional changes of the gonads were described [18]. Moreover, mutation of* Wnt4* gene in mouse causes ectopic expression of Leydig cells markers (e.g., 17-alpha-hydroxylase and 17-beta-hydroxysteroid dehydrogenase) [18]. Increased amounts of testosterone were also secreted [18].

The key role of the* WNT5A *gene has been documented by finding that* Wnt5a* knockout mice have no reproductive organs and live no longer than 24 hours [20]. Constitutively expressed* Wnt5a* gene was observed in mice gonadal ridges [21]. Wnt5a protein is present in the mouse endometrial stromal cells and its amount decreased in the area of myometrium formation what was established with the use of ribonuclease protection analysis and RNA* in situ* hybridization [20, 22, 23].

Female mice lacking* Wnt7a* genes expression have deformed wall of the uterus and undeveloped ovaries [6]. Moreover, it was shown that the expression of the Wnt7a may have impact on the transformation of Müllerian ducts [6].

### 3.2. Role of WNT Proteins in Endometrial Physiology

**Figure 1:**
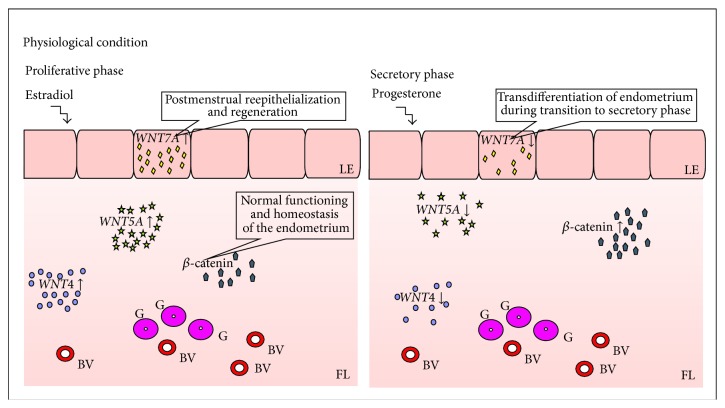
Endometrial expression of* WNT* genes. LE: luminal epithelium; FL: functional layer; BV: blood vessel; G: glands; ↓: decreased gene expression; ↑: increased gene expression.

In physiological condition (Figure 1),* WNT4* gene expression is higher in endometrial stroma in comparison to its expression in epithelial cells [12]. Injection of estradiol (E_2_) into ovariectomized mice upregulated, while administration of progesterone (P_4_) had no effect on,* Wnt4* gene expression in stromal cells of endometrium [24]. Similar pattern of expression as those of* Wnt4* was presented by* Wnt5a* during the luteal phase of the estrous cycle in mice [23] but expression of* Wnt5a  *was observed only in stromal cells just before and soon after estrus occurrence [23]. However, other authors could not confirm these findings [24]. Treatment of cyclic ewes with P_4_ and antagonist of P_4_ receptor at the same time (RU 486) increased endometrial* WNT5A* mRNA level at day 12 of pregnancy [25].

Fan and coworkers [26] showed that* WNT7A* mRNA levels in the female endometrial tissue were higher in the proliferative phase in comparison to secretory phase of the menstrual cycle. However, other authors did not find correlation of the* WNT7A* gene expression with phases of the menstrual cycle with the use of real-time PCR, digoxigenin-labeled cRNA probes, and* in situ* hybridization technique [11, 27, 28]. Presumably,* WNT7A *expression is stimulated by estradiol [29] which coordinates WNT7A-mediated process of postmenstrual reepithelialization and regeneration of the endometrium [26]. The influence of estradiol on* WNT7A* gene expression was presented in* in vitro* culture of luminal epithelial cells of human endometrium [30] or neonatal piglets [10]. Presence of WNT7A was marked in regenerating newly formed surface epithelium and upper endometrial glands [11, 26–28] but not in the lower glands and stroma of human endometrium [26, 28]. These observations support the view that luminal epithelium secretes factors that are important for glandular function and stromal transformation [11]. Moreover, progesterone-mediated downregulation of* WNT7A* gene expression may be essential for the transdifferentiation of endometrium during its transition to the secretory phase [26]. In mice,* Wnt7a* gene expression was completely suppressed in the surface epithelium and was undetectable in glandular epithelium and endometrial stroma after seven days of progesterone treatment [26].

**Figure 2:**
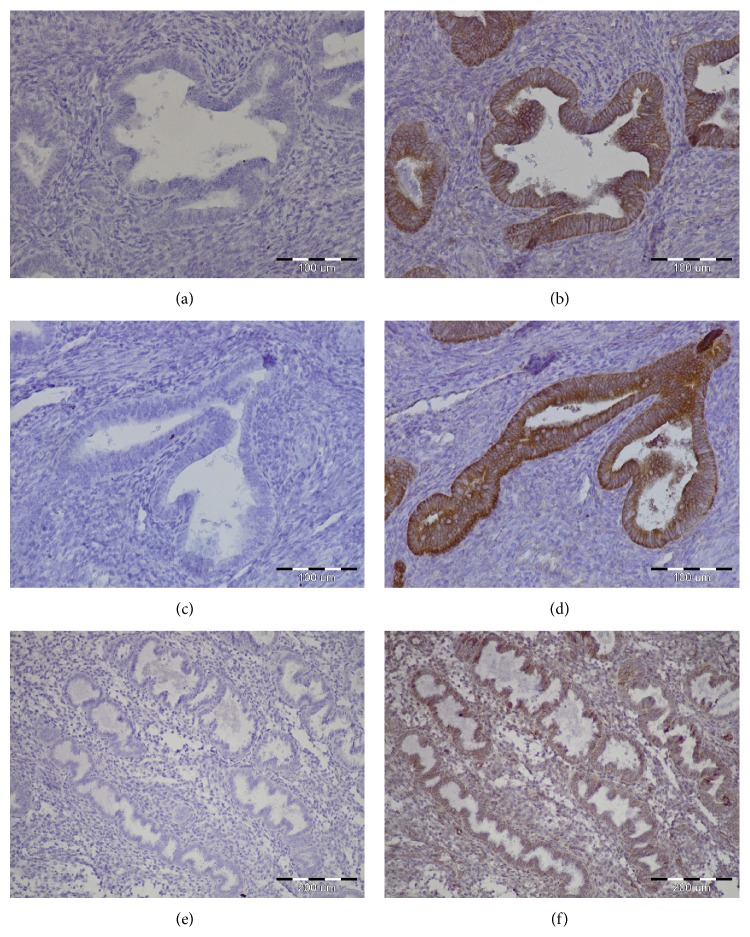
Examples of immunohistochemical staining confirming presence of *β*-catenin in physiological endometrium. Positive staining (BD Transduction Laboratories Cat. number 610153) of endometrial glands, magnification ×20 (b, d) and ×10 (f). Negative controls magnification ×20 (a, c) and ×10 (e).

*β*-catenin, the mediator of Wnt/*β*-catenin signaling, was first isolated as an intracellular protein constituting the binding domain of E-cadherin with cell's cytoskeleton [31]. The available data suggest that *β*-catenin is essential for normal functioning of the uterus and seems to be responsible for establishing of the endometrial homeostasis [32]. In human endometrial tissue, the immunoreactivity of *β*-catenin was observed in intercellular borders of luminal and glandular epithelial cells as well as in stroma and endothelial cells [33]. Examples of *β*-catenin positive staining of physiological endometrium are presented in Figure 2.

Fujimoto and coworkers [34] revealed upregulation of *β*-catenin (*CTNNB1*) mRNA level during secretory phase in human endometrium. It correlated with steroid hormone profile because progesterone but not estradiol increased* CTNNB1* mRNA level in human endometrial stromal cells cultured* in vitro* [35]. During proliferative phase of the menstrual cycle, the amount of nuclear *β*-catenin increased. *β*-catenin was allocated from the nucleus to the cytoplasm and cell membrane during the secretory phase [36]. However, Tulac and coworkers [11] showed no statistically significant difference in* CTNNB1* gene expression in human endometrium between proliferative and secretory phases.

Moreover, usage of LiCl, potential inhibitor of WNT/*β*-catenin signaling, induced estradiol-mediated proliferation and hyperplasia of endometrial cells in mice [37] and humans [38]. Activation of WNT/*β*-catenin signaling pathway increased endothelin 1 mRNA level which is target gene of *β*-catenin action as well as participant of endothelial cells differentiation [39]. Thus, it may be concluded that Wnt/*β*-catenin signaling regulates processes of endometrial proliferation and differentiation. Specific pattern of* WNT* genes expression and pattern of hormonal regulations are summarized in Table 1.

## 4. *WNT* Protein and Gene Expression in Endometrial Cancer

### 4.1. General Characteristics of Endometrial Cancer

Endometrial cell carcinomas (ECCs) are the most common malignancy of the female genital tract in the Western world and the fourth most common one after breast, lung, and colorectal cancer in women. A constant increase of endometrial cancer has been observed in the recent years [40]. ECCs occur mainly in postmenopausal women at the age of 55–65. Factors increasing the probability of the endometrial carcinoma occurrence include long-term estrogen therapy, polycystic ovarian syndrome, a history of nulliparity or infertility, irregular menstrual cycles, obesity, diabetes mellitus, and hypertension [41]. The curability of EECs is as high as five-year survival rate for the G1 and 1st stage is 90%. However, 5-year survival rapidly decreases to 30–50% for the 2nd and to 20% for the 3rd stage [41]. Women with ECCs experience dysfunctional endometrial bleeding which makes tumor detection seemingly an easy task. However, major diagnostic and prognostic problems often arise by histopathological assessment (WHO classification) since seven types of endometrial carcinoma can be distinguished. The most common subtype of ECC is an endometrial endometrioid adenocarcinoma (EEAC), classified as type I or estrogen-dependent cancer [42]. Approximately 80% of newly diagnosed endometrial carcinomas in the Western world are of the endometrioid (EEAC) type [43]. Any factor that increases exposure to unopposed estrogen (estrogen-replacement therapy, obesity, anovulatory cycles, and estrogen-secreting tumors) increases the risk of these tumors, whereas factors that decrease exposure to E_2_ or increase P_4_ levels (oral contraceptives, smoking) tend to be protective [44]. Type I endometrial cancer consists of low-grade endometrioid histology, starts with the background of endometrial hyperplasia, and may have better prognosis [41]. Endometrial serous adenocarcinoma (ESC) and clear cell endometrial carcinoma (ccEC) are aggressive neoplasia carrying a poor prognosis [45]. ESC or ccEC is estrogen-independent and is classified as type II [42]. The average age of patients with nonendometrioid cancer is 67 years, and at least half of them had cancer already spread beyond the corpus of the uterus at the time of diagnosis. The 5-year survival is approximately 62% for clear cell carcinomas and 53% for papillary serous cancers [44]. Although the ECCs are highly curable, there are particular morphological variations and histopathological features which do not allow for their clear identification [42]. Each subtype has specific genetic alterations showing microsatellite instability and mutations in* PTEN*,* PIK3CA*,* K-ras*, and* CTNNBI* (*β-catenin*) genes summarized in Table 2. However, their specificity as a biomarker has been widely discussed [42].

### 4.2. *Wnt* Gene Expression in Endometrial Cancer

**Figure 3:**
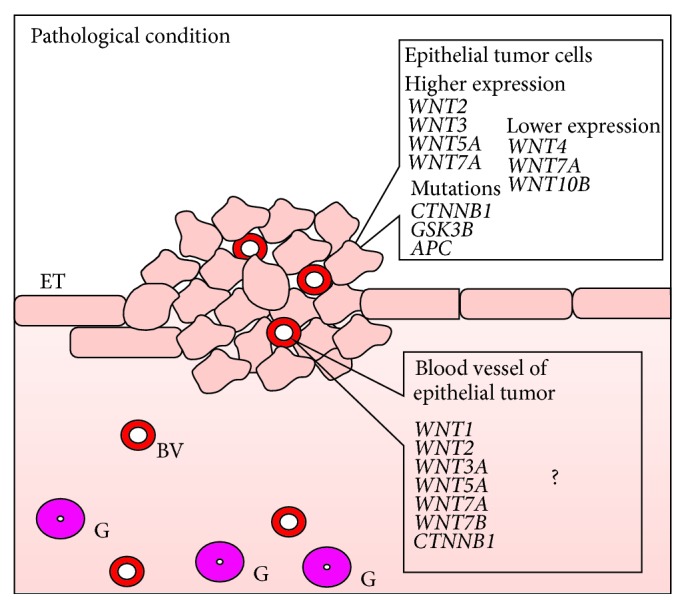
Expression of* WNT* genes in endometrial cancer. ET: epithelial tumor; BV: blood vessel; G: glands.

The expression of genes encoding WNT proteins and proteins involved in WNT signaling pathways was found to be changed also in endometrial cancer [12] (Figure 3). The* WNT4* mRNA level was lower while* WNT2*,* WNT3*, and* WNT5A* mRNA levels were higher in endometrial carcinoma in comparison to normal endometrium [12]. Also* WNT2*,* WNT3*,* WNT4*, and* WNT5A* genes expression was higher in normal human primary epithelial and stromal endometrial cultures compared to endometrial carcinoma cell lines, what suggest their participation in endometrial neoplasia [12].

Most of the studies concentrated on* WNT7A* gene expression. In 63% patients of one series of endometrial carcinoma,* WNT7A* gene expression was absent or reduced and negatively correlated with FIGO stage, grade, lymph node metastasis, depth of myometrial invasion, lymph vascular space involvement, and peritoneal cytology [28]. In large-scale population study on 244 EEC patients,* WNT7A* overexpression was found in most cases of endometrial cancer in comparison with normal endometrium and benign endometrial lesion [46]. However, negative expression of* WNT7A* gene correlated positively with overall survival and disease-free survival of endometrial cancer [46].

In the Ishikawa cell line model of endometrial adenocarcinoma, estrogen receptors were probably involved [47] in the downregulation of* WNT7A* expression mediated by estradiol [48]. Moreover,* WNT7A* and* WNT7B* genes expression was increased in endometrial carcinoma cell lines and normal endometrial tissues as compared with primary cultures of human endometrial cells [12].

WNT10A and WNT10B proteins have been implicated in estrogen-related carcinogenesis of endometrial cancer. The amount of WNT10B protein was higher in endometrial cancer than in hyperplastic and normal endometrium as determined by Western blot technique [49]. In early stages of endometrial cancer, the expression of* WNT10B* was higher than in later stages. WNT10B proteins were mainly detected in patients with the cancer of endometrioid type, who had high graded and advanced-staged tumor without lymph node metastasis [49]. This clinical study was partially confirmed by results of the* in vitro* investigations.* WNT10A* gene expression was decreased in endometrial HEC1B and AN3CA cell lines while WNT10B was increased in Ishikawa cell lines [50].

**Figure 4:**
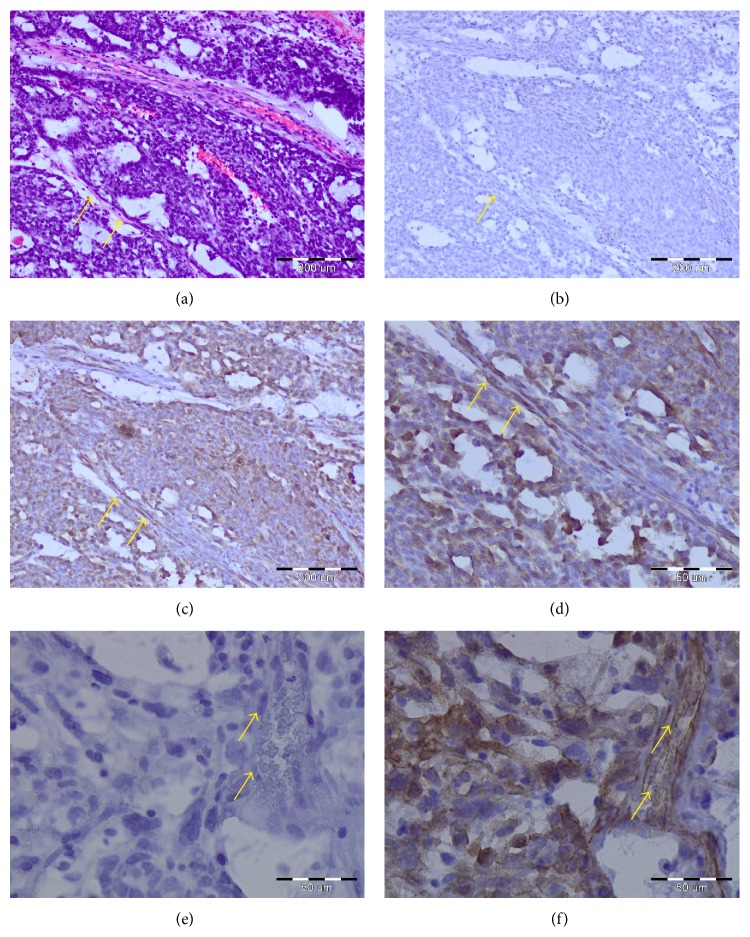
Examples of immunohistochemical staining confirming presence of *β*-catenin in adenocarcinoma endometrioid (G3) cells and tumor vascular endothelial cells (arrow). Hematoxylin-eosin staining (a); negative controls in magnification ×20 (b) and ×40 (e); positive staining (BD Transduction Laboratories; Cat. number 610153) in magnification ×20 (c) and ×40 (d, f).

Mutations of *β*-catenin gene (*CTNNB1*) were found in many endometrial cancers [51]. According to various reports, 10 to 45% of endometrial cancers present missense mutation of* CTNNB1* [50]. Endometrial tumors with mutation in exon 3 on serine/threonine residue showed predominant nuclear *β*-catenin accumulation. In this case, blockage of the process of *β*-catenin degradation results from the lack of its phosphorylation [36, 52, 53]. In rat gliomas [54], human glioblastomas [55], and medulloblastomas [56], nuclear accumulation of *β*-catenin was observed in endothelial cells of neovessels. Beta-catenin accumulation was observed in the nucleus of malignant changed endometrial cells [36, 52, 53]. However, as far as we know the presence of *β*-catenin was previously not shown in tumor vascular endothelial cells (Figure 4). However, *β*-catenin membranous immunoreactivity associated with E-cadherin decreased during transformation of normal endometrium through atypical endometrial hyperplasia to endometrial cancer in parallel with decreased E-cadherin expression in endometrial cancer [57].* CTNNB1* mutation is observed mainly in endometrioid endometrial cancer [57–59].

Mutations in* KRAS* and/or* CTNNB1*,* GSK-3β*, and* APC* gene are recognized as major alterations in type I endometrial cancer [50]. Upregulation of estrogen receptor signaling causes endometrial hyperplasia and can be a reason of endometrial cancer [29]. WNT signaling activation leads to endometrial and myometrial hyperplasia [32, 60, 61], squamous cell metaplasia without malignant transformation [62], mesenchymal tumors, and endometrial sarcomas [32, 61] in transgenic mouse [62].

DKK1 was highly expressed in benign endometrial tissue and downregulated in endometrial cancer [63]. Treatment of Ishikawa cell line with DKK1 lowered the level of active *β*-catenin as the result of Wnt signaling pathway inhibition through binding to LRP5/6 [63]. DKK1 is positively correlated with histological differentiation and clinical stage of endometrial cancer [63].* DKK3*  gene expression was found to be decreased in endometrial cancer. It correlated with advanced stage and high risk clinicopathological factors [51]. High expression of* Dkk3* gene reduced motility and proliferation of the cells in* in vitro* experiments [51].

## 5. Crosstalk of WNT Proteins and Other Factors in Endometrial Angiogenesis 

### 5.1. Angiogenesis in Endometrium

Vascular system is a network of arteries, capillaries, and veins for transport of gases and macromolecules. Vasculogenesis is a process of* de novo* formation of capillary bed through differentiation, proliferation, and migration of precursor cells (angioblasts) [64]. Formation of new blood vessels from already existing capillaries is called angiogenesis [65]. Angiogenesis is a two-step, physiological process essential for proper endometrial functioning [66]. Blood vessels have to be repaired after menstrual phase of the menstrual cycle [66]. Capillaries grow, mature, and coil during the proliferative and secretory phase [66], when endometrial blood flow and permeability of endometrial microvessels become rapidly increased by high levels of estrogens at the late phase of cycle [67]. It is highly probable that vessel growth in human endometrium occurs by nonsprouting mechanism, elongation in response to metabolic demands of surrounding cells [68] and intense hypoxia in the luminal portion of the endometrium on day 2 of the cycle, with negligible detection by d5 [69]. Endothelial cells which form capillary bed are under influence of (i) factors produced by surrounding tissue [65] and/or (ii) angiogenic factors that circulate in blood and their levels fluctuate during menstrual cycle [70]. Growth factors (VEGF, EGF, FGF, NP-1, and angiopoietin) and their receptors (VEGFR, EGFR, FGFR, and IGFR) can both positively or negatively influence this process. The common denominator for the process of angiogenesis occurring in the endometrium is hypoxic environment and hypoxia inducible factor (HIF) stimulation of VEGF expression [71].

### 5.2. Participation of WNT Proteins in Blood Vessel Formation

Participation of WNT proteins in the process of differentiation of cells of hematopoietic and endothelial cell lineage as well as vasculogenesis and angiogenesis is apparent [72]. It has been demonstrated that sustained WNT pathway activation can be utilized to generate endothelial progenitors from mesodermal lineage of embryonic stem cells in* in vitro* conditions [73]. Moreover, coculture of human embryonic stem cells with* Wnt1*-overexpressing cells accelerated differentiation of mesoderm germ layer into hematoendothelial cells* via* activation of canonical WNT signaling [74]. Presence of WNT1 upregulated WNT/*β*-catenin signaling, bovine aortic endothelial cells proliferation, and capillary stability under* in vitro* conditions [75]. On the other hand, WNT1 was found to inhibit proliferation of endothelial cells [76].

The role of the WNT2 protein in angiogenesis is less clear. Increased expression of WNT2 protein and FZD-5 receptor caused defects in the vasculature of murine placenta and changed blood flow in the mouse yolk sac [77, 78] as a reduced number of the fetal capillaries were observed [79]. However, expression of* WNT2* gene had no impact on WNT/*β*-catenin signaling activation, endothelial cells proliferation [72, 75], or capillary length [75]. Differentiation of endothelial cells from mouse embryonic stem cells is suspected to be controlled by Wnt2 and Wnt11 [39].

WNT3A was shown to be direct, VEGF-independent, inducer of endothelial cell proliferation [72, 80] and migration [80].

WNT5A is required for endothelial differentiation of embryonic stem cells and transformation of mouse embryonic stem cells into immature endothelial progenitor cells, taking part in healing process of damaged endothelium [39]. Acting on endothelial cells through autocrine regulation [81], WNT5A can decrease cell number and capillary length. However, these effects were not observed after activation of WNT/*β*-catenin signaling [75]. Moreover, WNT5A protein did not stimulate human umbilical vein endothelial cells (HUVEC) migration and proliferation [75]. Wnt5a and Wnt10b induced FZD-5-mediated angiogenesis in a mice yolk sac [79].

WNT7 proteins were shown to promote normal angiogenesis in ventral regions of the CNS in mouse [82]. Specifically* Wnt7a* but not VEGF promotes migration and stimulates expression of blood-brain barrier specific transporters of glucose (GLUT-1) in mouse brain endothelial cell line [82]. Moreover, in* Wnt7b* gene deficient mice, loss of* Wnt7b* gene resulted in defective smooth muscle component of the major pulmonary vessel differentiation, degradation of vessel's wall, and perinatal hemorrhage [83].

Inhibition of the expression of* CTNNB1* gene in endothelial cells affected the formation of vasculature of head of mouse embryos, large vitelline and umbilical vessels, and the vasculature of the placenta [84]. As a result of *β*-catenin absence, significant reduction in cell junctions organization and hemorrhage was observed [84].

### 5.3. Hypothetical Involvement of the Wnt/*β*-Catenin Pathway in Endometrial Angiogenesis

Data provided in the proceeding chapter clearly indicate the participation of WNT proteins in the recruitment, proliferation, and migration of endothelial cells in healthy subjects and cancer patients. In the endometrium, development of the vascular network occurs simultaneously with epithelial and stromal cells expansion, expression, and influence of angiogenic factors that circulate in blood and fluctuate in menstrual cycle phase-dependent manner [70]. Regulation of endothelial cell growth and fate was shown to be regulated by reciprocal interactions between mesenchymal and endothelial cells [81]. Even if estradiol was shown to inhibit angiogenesis under* in vivo* conditions [85], this effect was not caused by direct action on endothelial cells because they do not have estrogen receptors [86]. Therefore, they cannot respond to this potential inhibitor of angiogenesis. Thus, it is highly probable that in endometrium capillaries are under influence of other external factors which compensate or antagonize the influence of estradiol. We suggest that WNT proteins are perfect candidate to be such a mediator.

**Figure 5:**
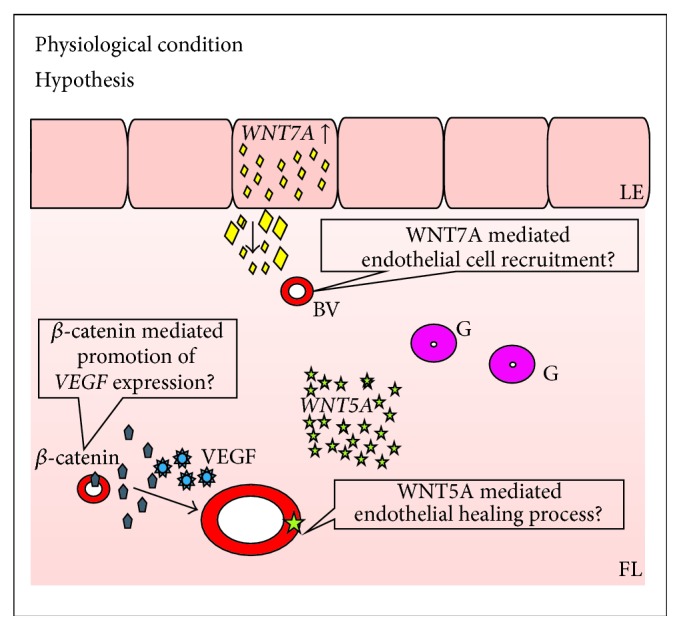
Hypothetical involvement of WNT and *β*-catenin proteins in endometrial angiogenesis. LE: luminal epithelium; FL: functional layer; ET: epithelial tumor; BV: blood vessel; G: glands.

We can hypothesize (Figure 5) that it might be probable that in physiological condition WNT5A can participate in endothelium recovery, rather than angiogenesis process, as it takes part in healing of the damaged endothelium [39], but not in proliferation and migration of the endothelial cells [81] or increasing capillary length [75]. WNT7A might be chemoattractant for endothelial cells in the process of physiological endometrial angiogenesis, as it is a factor of epithelial origin.* WNT7A* gene expression was upregulated during proliferative phase of the estrous cycle and downregulated in the secretory phase [26]. *β*-catenin can function in the endometrium either directly on endothelial cells or indirectly through its action on endometrial cells where it promotes the expression of VEGF [87] or endothelin 1 [39]. However, this hypothesis requires experimental confirmation.

## 6. Conclusions and Future Perspectives

The participation of WNT proteins, *β*-catenin, and inhibitors and inducers of WNT signaling in the process of endometrial angiogenesis is largely unknown. The main task which should be now undertaken should concentrate on defining which WNT, receptors, inhibitors, and signaling pathway are activated in the endothelial cells of the blood vessel of the endometrium. The spatiotemporal pattern of expression of elements of WNT signaling system should also be established. Differences in normal and pathological state should be also considered. Conducting this research will help to determine the angiogenic potential of the WNT family of proteins, will allow for a better understanding of the mechanism of formation of vessels in the endometrium, and will help to determine whether WNT proteins can be potential target for antiangiogenic therapy directed mainly against transcription factors as, for example, *β*-catenin. We are aware that our hypothesis does not present the experimentally verified information. However, we believe that our suggestion will contribute to the discussion and to some extent the development of research on the role of WNT proteins in the formation of blood vessels in the dynamically changing uterus.

## Figures and Tables

**Table 1:** Function and hormonal regulation of WNT and *β*-catenin (*CTNNB1* gene) in the endometrium.

Gene		Description	References
*WNT4 *	Function	Formation of the primordial gonads	[18]
		Gametogenesis	[17]
		Uterine wall morphogenesis	[19]
		Decidua formation	[24, 60, 88]
	Hormonal regulation	E_2_ ↑	[13]
		E_2_ ↓	[9, 10]
		P_4_	[13]
		E_2_ ↓ + P_4_	[13]

*WNT5A *	Function	Uterine wall morphogenesis	[19]
	Hormonal regulation	E_2_ ↓	[9, 10]
		P_4_ + RU486 ↑	[25]

*WNT7A *	Function	Development and functioning of the gonads	[6]
		Postnatal uterine gland morphogenesis and function	[89]
	Hormonal regulation	E_2_ ↓	[9, 10]
		E_2_ + P_4_ ↓	[24]

*CTNNB1 *	Function	Normal functioning of the uterus Endometrial homeostasis	[32]
	Hormonal regulation	P_4_ ↑	[35]
		E_2_	[35]

↓: decreased gene expression; ↑: increased gene expression; E_2_: estradiol; P_4_: progesterone, RU486: antagonist of progesterone receptor.

**Table 2:** Immunohistochemical and molecular markers for ECCs classification.

Method	Type of EEC				References
	Normal	EEAC	ESC	CcEC	
IHC	PTEN	PTEN ↓	PTEN −	PTEN −	[90]
	Active *β*-catenin	Active *β*-catenin +	Active *β*-catenin −	Active *β*-catenin −	[91]
	p53	p53 −/+	p53 +	p53 +	[92]

Real-time PCR	PTEN	PTEN ↓	PTEN	PTEN	[90]
	Survivin	Survivin ↑			[92]
	K-ras	K-ras ↑	K-ras ↓	K-ras ↓	[93]
	p27	p27 ↓			[92]

+: protein is present; −: protein is absent; −/+: protein is expressed moderately.

↓: decrease of gene expression; ↑: increase of gene expression.
